# Observation-driven generation of texture maps depicting dust accumulation over time

**DOI:** 10.1007/s00371-022-02457-7

**Published:** 2022-04-02

**Authors:** Rebecca L C Santos, Gladimir V G Baranoski

**Affiliations:** University of Waterloo, Waterloo, Canada

**Keywords:** Computer graphics, Realism, Texture, Material appearance, Weathering phenomena, Dust

## Abstract

The perception of realism in computer-generated images can be significantly enhanced by subtle visual cues. Among those, one can highlight the presence of dust on synthetic objects, which is often subject to temporal variations in real settings. In this paper, we present a framework for the generation of textures representing the accumulation of this ubiquitous material over time in indoor settings. It employs a physically inspired approach to portray the effects of different levels of accumulated dust roughness on the appearance of substrate surfaces and to modulate these effects according to the different illumination and viewing geometries. The development of its core algorithms was guided by empirical insights and data obtained from observational experiments which are also described. To illustrate its applicability to the rendering of visually plausible depictions of time-dependent changes in dusty scenes, we provide sequences of images obtained considering distinct dust accumulation scenarios.

## Introduction

**Fig. 1:**
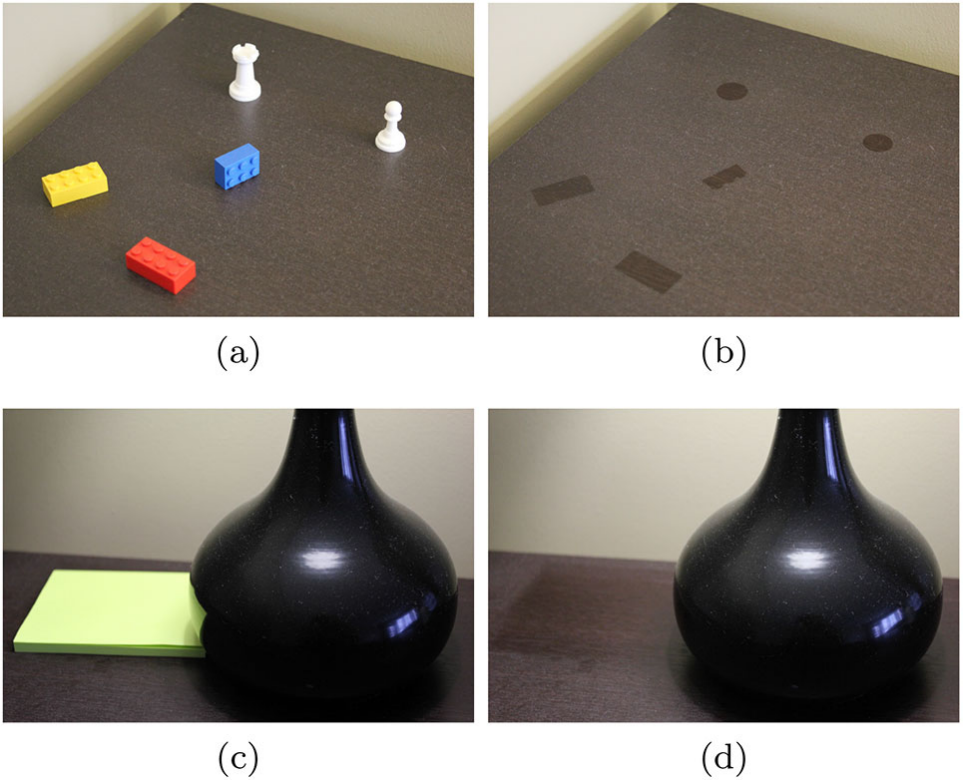
Photographs of real settings depicting the accumulation of dust over a period of several months. (**a**, **c**): photographs taken before the removal of objects from the settings. (**b, d**): photographs taken after the removal of the objects

Despite significant advances in realistic image synthesis in recent years, notably involving the simulation of weathering and aging phenomena (e.g. [5, 31, 33, 36, 40]), computer-generated images are often characterized by a “clean look”. Real settings, however, have a less pristine appearance. This can be perceived, for example, through visual cues resulting from time-dependent phenomena such as the accumulation of dust (Fig. 1), the focal point of this work. By accounting for the presence of this pervasive material and the temporal variations in its appearance, one can considerably improve the degree of realism of synthetic settings.

In order to effectively incorporate the presence of dust in synthetic settings, one should select an appropriate level of abstraction to represent its intrinsic complexity. For example, household dust can be composed of a wide range of materials, from textile fibres and hair strands to human skin fragments and mineral particles, just to name a few [8]. Moreover, the location of a household dust deposit can also affect its composition, notably in areas prone to variations in indoor environmental conditions ( e.g. ventilation changes [44]). Ideally, the selected level of abstraction should match the requirements of the rendering application in which it will be used.

A number of works addressing the rendering of dusty scenes have been presented in the literature ( e.g. [22, 25, 43]). To correctly simulate the appearance of dust using physically based approaches, one needs to correctly simulate complex light interactions with its distinct constituent materials. This often requires precise information about their spatial and size distribution patterns. Although such approaches may result in predictive images [21], they can potentially incur relatively high computational costs. Moreover, their effectiveness may be limited by the scarcity of measured optical and morphological data associated with the large variety of dust’s constituent materials.

Physically inspired approaches, on the other hand, are aimed at the generation of believable images. Although such images are not meant to be predictive, they can provide plausible representations of a given setting without one having to carry out potentially costly light transport simulations. Thus, although they may have a limited use in scientific applications requiring predictability, they are often adequate for applications with educational or entertainment purposes. It is worth noting that physically inspired (instead of physically based) methods are extensively employed in different areas of computer graphics (e.g. [4, 37, 41, 46]). These methods, albeit inspired by real physical observations, are developed from a relaxation of physically based methodology in order to avoid complex formulations and, consequently, keep the representation of different materials plausible at a relatively low cost. For the same reasons, we also elected to employ a physically inspired approach in our work.

In this paper, we present a novel framework for the generation of textures to be employed in the depiction of temporal changes in the appearance of dusty objects placed in indoor settings. The design of its core algorithms, whereas building on methodology adopted by existing physically inspired approaches, was driven by actual observations of dust accumulation over time. This strategy enabled us to strive for a high degree of plausibility while keeping the associated computational costs relatively low and the algorithms’ incorporation into existing rendering systems straightforward.

Accordingly, for the purposes of the proposed framework, we categorize dust’s composition into two roughness levels, one associated with an opacity film (formed by fine dust particles) and the other with a granularity mask (containing coarse dust elements like particle clumps and hairy structures). These abstraction levels, which are based on methodical visual examinations of deposited dust, are represented by textures. During rendering, these textures are combined taking into account the dependence of dust appearance on viewing and illumination geometries. The time-dependent specification of textures is supported by data collected from observational experiments on dust accumulation, which have been carried out over extended periods of time. To the best of our knowledge, rendering algorithms that simultaneously account for different levels of dust roughness and their temporal variations have not been presented in the computer graphics literature to date.

The remainder of this paper is organized as follows: In Sect. 2, we briefly present related works on the accumulation and rendering of dust. In Sect. 3, we highlight relevant aspects associated with the perceived appearance of accumulated dust. In Sect. 4, we outline the procedures employed to obtain supporting data on dust accumulation over time. In Sect. 5, we describe the proposed framework’s texture generation algorithms. In Sect. 6, we illustrate its applicability to realistic image synthesis and concisely address possible extensions to its formulation. Finally, in Sect. 7, we conclude the paper and point out directions for future research related to this topic.

## Related work

In this section, we concisely review relevant publications that have specifically addressed the accumulation and rendering of dust. It is worth noting that there is a large body of work on topics that could be connected to the research presented in this paper. Besides the simulation of weathering and aging phenomena mentioned earlier, these topics include the modelling and rendering of granular materials, such as snow and sand (e.g. [6, 18, 29, 30, 35], as well as the simulation of dust dynamics (e.g. [3, 10, 12, 13, 26]), among several others. A comprehensive review of the literature on these topics, however, would merit an entire publication devoted to it, which is beyond the scope of this work.

### Dust accumulation

Hsu and Wong [25] proposed an algorithm to simulate the accumulation of dust that employs the dot product of a target surface’s normal vector and the normal vector of a user-defined dust source to determine the amount of deposited material. They also considered external factors that may affect the amount such as scraping off by other objects. The dust accumulation patterns resulting from the application of their algorithm are stored as texture maps. Lu et al. [33] indicated that the algorithm proposed by Hsu and Wong [25] could be combined with other techniques, notably ambient occlusion, to obtain physically plausible context-aware textures depicting the presence of dust.

Afterwards, Chen et al. [15] proposed an algorithm for handling material lifespan changes leading to rusty and dusty appearances. They also made use of the target surfaces’ normal vector, while incorporating additional weathering concepts such as user-defined material deterioration functions.

More recently, Guo and Pan [22] introduced an algorithm based on the application of shadow mapping and ambient occlusion techniques to determine the amount of dust at each point on a target surface. We note that the dust accumulation patterns resulting from the application of these algorithms are relatively uniform, i.e. certain roughness aspects, such as the presence of dust particle clumps and fibrous (hairy) structures, are not normally explicitly incorporated into the simulations.

### Dust rendering

Once the dust has been placed within a scene (e.g. deposited on the surface of an object), it must be rendered. Most of the algorithms employed in the rendering of accumulated dust can be classified as *ad hoc*, i.e. they are created and used for that particular purpose. These algorithms usually rely on the use of texture maps and noise functions. Accordingly, individual dust particles and particle clumps are seldom explicitly accounted for. Although these algorithms are not intended to follow a physically based approach, they can produce believable results without requiring extensively time-consuming computations.

For instance, in the work by Hsu and Wong [25], the user must define materials at two extremes, namely covered by dust and devoid of dust. From there, the algorithm linearly interpolates between these two extremes according to the amount of dust calculated previously. Hsu and Wong [25] also suggested that the appearance of the rendered dusty materials could be improved by adding Perlin noise [39].

Similarly, Chen et al. [15] proposed an algorithm that relies on a user-defined custom deterioration function (e.g. involving changes in the diffuse and specular components of a material’s reflective behaviour). This function is then incorporated into the user’s shading method of choice. Also, Ashraf and Chong [3] used a 3D fractal surface shader to imitate a thin layer of dust.

Adachi et al. [1] modelled thick layers of dust through the combined use of parametric shell textures and Perlin noise [39]. In contrast to the previously cited works, their algorithm is focused on the fibrous component of those layers. It is worth mentioning that, despite the fact that many of these *ad hoc* algorithms rely on the availability of suitable texture maps, methods to obtain physically plausible dust textures, such as those proposed by Hoock [24] and Ofek et al. [38], are still scarce in the literature.

Although physically based approaches specifically tailored to the rendering of dusty materials have not been extensively explored by the computer graphics community, there are noteworthy initiatives in this area. For example, building up on the algorithms presented by Blinn [7], Sun et al. [43] proposed a framework for the acquisition and rendering of time-varying changes in the reflective behaviour of different materials, including dusty surfaces. Guo and Pan [22], on the other hand, employed a model based on the Kubelka–Munk theory [28, 32] to render settled dust. Again, we note that these physically based approaches also resulted in relatively uniform representations of a layer of accumulated dust.

**Fig. 2:**
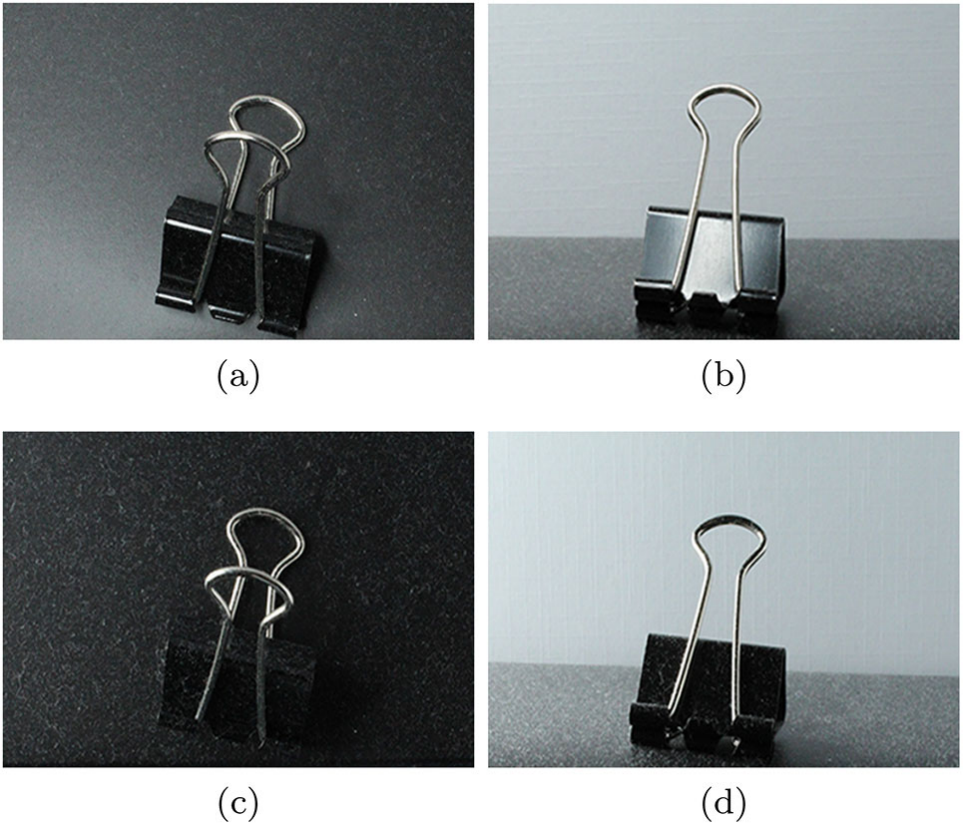
Photographs of a dusty scene depicting the visual effects of variations in the viewing and illumination geometries. The photographs were taken considering distinct viewing ($\theta _v$) and light incidence ($\theta _i$) angles measured with respect to flat surface’s normal vector. **a** $\theta _v=0^{\circ }$ and $\theta _i=0^{\circ }$. **b** $\theta _v=80^{\circ }$ and $\theta _i=0^{\circ }$. **c** $\theta _v=0^{\circ }$ and $\theta _i=80^{\circ }$. **d** $\theta _v=80^{\circ }$ and $\theta _i=80^{\circ }$

## Empirical background

We remark that the algorithms proposed in this paper are based on insights obtained from careful visual assessments of real dusty settings. In this section, we present observed qualitative characteristics that provided the basis for the selection of the distinct levels of abstraction used to characterize a dust layer in this work, namely the opacity film and the granularity mask. Recall that we employ the former to represent fine dust particles, and the latter to represent coarse dust elements such as particle clumps and hairy structures.

Although our observations involved a number of distinct settings, for conciseness and without loss of generality, we selected a representative case to be used as an observation reference in this section. It consists of a paper clip on a flat surface as depicted in the photographs presented in Fig. 2. More precisely, we use this setting to verify how the perception of the different levels of dust roughness is affected by variations on the viewing and illumination geometries, which have as their main parameters of the viewing angle (denoted by $\theta _v$) and the light incidence angle (denoted by $\theta _i$), respectively. For consistency, both angles are measured with respect to the flat surface’s normal vector.

**Fig. 3:**
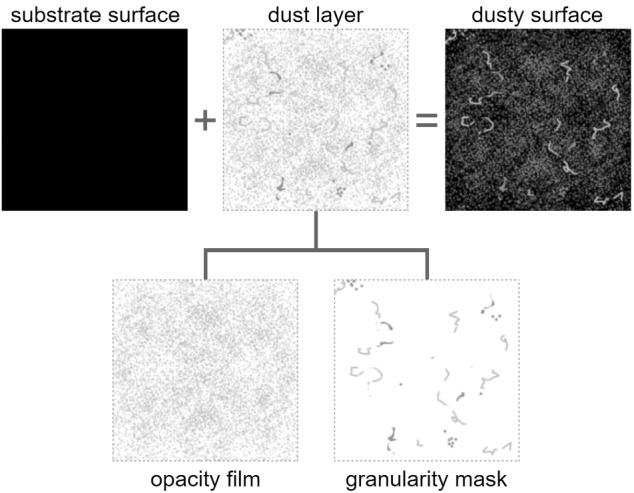
Diagram illustrating the contributions of fine and coarse dust components, respectively, represented by an opacity film and a granularity mask, to the appearance of dusty surfaces

As shown in the photographs presented in Fig. 2a, c, large-scale features, such as particle clumps and hairy structures, are clearly noticeable upon a top view ($\theta _v=0^{\circ }$) inspection of the scene, while fine dust particles are less prominent. As the viewing angle is increased to enable a grazing view ($\theta _v=80^{\circ }$) inspection of the scene, the film of fine dust particles becomes more noticeable as shown in Fig. 2b, d, while variations in the perceived appearance of large-scale features are negligible for practical purposes.

Recall that the photographs presented in Fig. 2 were obtained considering two light incidence angles, $\theta _i=0^{\circ }$ and $\theta _i=80^{\circ }$. By considering a large light incidence angle, which corresponds to the placement of the light source in a grazing position, one can make both levels of dust roughness more noticeable. For instance, one can observe more clearly the relatively nonuniform shape of the particle clumps and hairy structures in Fig. 2c (in comparison with Fig. 2a), as well as the overall presence and light reflective properties of the film formed by fine dust particles in Fig. 2d (in comparison with Fig. 2b).

**Fig. 4:**
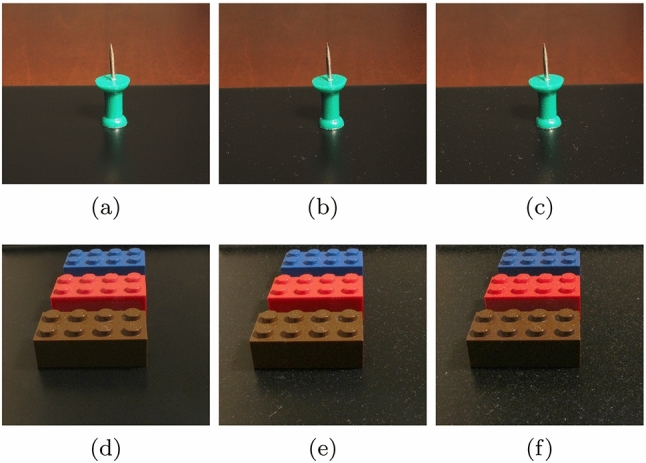
Photographs depicting two real settings employed in our observational experiments on dust accumulation over time. The thumbtack scene (top row) was set in a room with a low level of human presence and activity, while the lego scene (bottom row) was set in a room with high level of human presence and activity. **a** and **d**: initial state. **b** and **e**: after 10 days of dust accumulation. **c** and **f**: after 20 days of dust accumulation

Clearly, the appearance of a dusty surface depends on all of the different levels of dust roughness. However, our observations indicate that, for analysis and rendering purposes, a dust layer can be virtually decomposed into the two abstract representations employed in this work, namely the opacity film and the granularity mask, as illustrated by the diagram presented in Fig. 3. Moreover, these observations also indicate that the contributions of these two representations need to be properly modulated, by taking into account the viewing and illumination geometries employed in a given scene, in order to realistically depict the appearance of dusty surfaces. These aspects are accounted for in the formulation of the algorithms proposed in this work (Sect. 5).

## Dust accumulation experiments

Besides the aspects outlined in the previous section, we also carried out a number of observational experiments to obtain additional supporting information for the design of the proposed texture generation framework (Sect. 5). In the remainder of this section, we briefly describe the procedures used to gather and analyze these datasets.

Again, for conciseness and without loss of generality, we present here two representative cases of our observational experiments. They involved the daily photographic record of dust deposited in two distinct real-life settings (Fig. 4). Although we have initially considered a temporal resolution in terms of hours instead of days, we found that the latter allows us to obtain more meaningful insights since hourly variations on dust accumulation are normally negligible upon visual inspection.

The analysis of the resulting photographs, which involved the quantification of visually relevant dust components (fine particles and coarse elements), present in each setting on each day, provided us with a baseline for the design of the algorithms employed by the proposed framework. More explicitly, we were able to obtain approximated time-dependent descriptions of the dust accumulation processes. These descriptions, in turn, were employed to guide the generation of texture representing accumulated dust in synthetic scenes at different time instances (Sect. 5).

The selected settings, which are shown in Fig. 4, included distinct objects placed in environments with distinct levels of human presence and activity so that we could increase our scope of observations. The central positioning of the objects (thumbtack and legos) on a flat dark surface enabled consistent focal length between photographs taken at different time instances. This surface was selected in order to enable a high degree of fidelity in the visual detection of dust elements. The thumbtack scene (Fig. 4a–c) was set in a room with a low level of human presence and activity, while the lego scene (Fig. 4d–f) was set in a room with high level of human presence and activity.

After taking the photographs, fine particles and coarse dust elements on the dark flat surface were marked and counted using standard image processing tools. This initially involved the generation of raster files consisting of small ellipses in the locations of each of the dust particles. To accomplish that, the images were processed using an image editing software (Adobe Photoshop [27]), and a transparent layer was created above each photograph. On this new layer, areas of the underlying photograph containing dust particles were marked with an ellipse. With the particles converted into ellipses, we were then able to count the markings within the final images using the *imfindcircles* MATLAB function [20], which relies on a circular Hough transform [45].

The number of coarse dust elements, such as relatively large clumps and hairy structures, was compounded to inform the generation of the granularity masks, whereas the number of fine particles was employed to inform the generation of the opacity films used by the proposed framework (Sect. 5). We remark that we chose to adopt a physically inspired approach in the design of the proposed algorithms. Accordingly, our observational experiments and the analysis of their outcomes were aimed at obtaining approximate estimates of the relative number of dust elements, rather than precise readings.

**Fig. 5:**
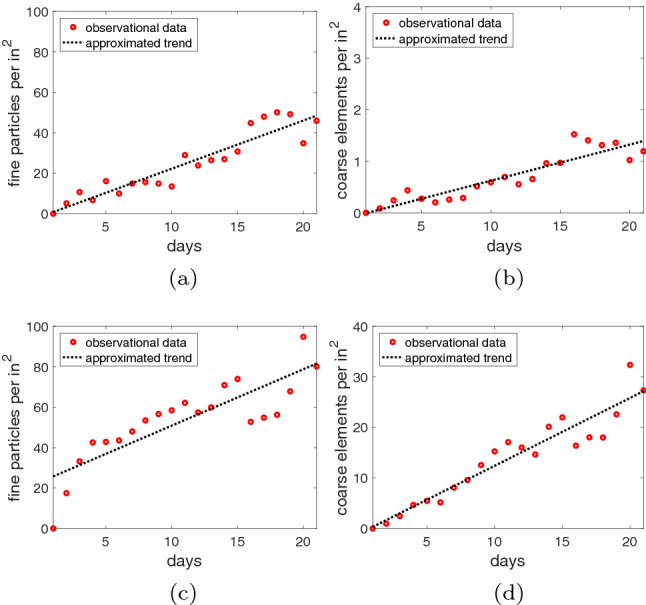
Graphs depicting the observational data, along with their respective approximated trends, obtained for the thumbtack (top row) and lego (bottom row) settings. **a** and **c**: Fine particle density over time. **b** and **d**: Coarse element density over time

To facilitate comparisons between the outcomes of the observational experiments associated with the two photographed settings, we employed a simple normalization procedure. It consisted in dividing the total numbers of marked dust elements for each day by the respective areas of the dark flat surface considered during the marking operation carried out for each setting. This, in turn, resulted in total dust element numbers normalized per $in^2$ (or 6.4516 $cm^2$) of the dark flat surface. The obtained values along with their respective approximated trends over time are shown in the graphs presented in Fig. 5. We note that the approximated trends, which are presented for illustrative purposes, were obtained using the least square method [9, 23].

The results depicted in the graphs presented in Fig. 5 indicate that although the number of fine particles and coarse elements oscillates in relatively short periods of time, they tend to increase in an approximate linear fashion in longer periods of time. We note that such oscillations, which may result from transient changes in air circulation inside a room, are usually perceptually inconspicuous when one takes glances at a dusty scene within a period of few successive days.

Note that the accumulation rate of fine dust particles in the lego scene depicted in Fig. 5c is steeper in the first five days than the rate observed in the subsequent days. Since we attempted to keep the environmental conditions constant during our experiments, this initial steeper rate may have been elicited by a slightly stronger level of human presence and activity in the room during the initial five days of the dust accumulation process. This suggests that different periods of dust accumulation within a given setting could be approximated by distinct linear functions in a piecewise fashion. We explore this aspect further in Sect. 6.

As can also be verified in the graphs presented in Fig. 5, the number of fine dust particles marked in a given day was consistently larger than the number of coarse elements. This was expected since some of these elements often result from the aggregation of many fine particles over time. Lastly, the density of particles and coarse elements was considerably higher in the lego scene set in a room characterized by a higher level of human presence and activity. This observation is in agreement with empirical evidence reported in the literature, indicating that human skin fragments represent one of the main sources of indoor dust [8].

## Proposed framework

In this section, we describe the proposed texture generation framework, whose design was guided by the qualitative and quantitative aspects addressed in Sects. 3 and 4. Throughout its description, we use vector (bold) notation to represent colours in RGB format. We note, however, that the application of the proposed framework can be extended to other colour formats.

We begin by outlining the general approach adopted for generating textures that represent the accumulation of dust over time. We then introduce the procedures to generate the textures for the opacity film and granularity mask at a given timestep. Lastly, we address the application of the generated textures to the rendering of dusty scenes.

### Overview of temporal aspects

While describing our framework, we focus on scenes in which dust naturally accumulates over time according to the level of human presence and activity. In other words, we consider that the process is not affected by sporadic disturbances (e.g. a wind gust or a mechanical intervention). We discuss how such disturbances can be potentially accounted for in Sect. 6.2.

For a scene with no sporadic disturbances, once a dust particle is placed at a location in a given timestep, one can assume that it will be present at the same location for the entire simulation. Following this assumption, one can represent the accumulation of dust at a given timestep by iterating through each of the preceding timesteps until all desired dust particles have been placed at their respective locations within the scene.

The outcomes of our observational experiments (Sect. 4) indicated that changes in the density of accumulated dust elements over time may be approximated by a linear relationship. Accordingly, we elected to calculate the number of dust elements to be *added* to a target object’s surface at a given timestep *t* (in days) as:

1 $$\begin{aligned} p(t) = {\left\{ \begin{array}{ll} A(\alpha + \beta ) &{} t = 1, \\ A\alpha &{} \text {otherwise}, \\ \end{array}\right. } \end{aligned}$$

where *A* represents the approximate surface area of the target object in $in^2$, and the parameters $\alpha$ and $\beta$ correspond to densities expressed in terms of the number of dust elements per unit of area ($in^2$). A large value for $\alpha$ would indicate a steeper rate of dust accumulation, which would be appropriate for settings characterized by a high level of human presence and activity. A large value for $\beta$ would indicate a high initial (after one day) amount of deposited dust elements.

### Opacity film

As indicated in Sect. 3, the opacity film consists of fine dust particles. We represent the appearance of this film as a texture map (raster file) to be used in the final rendering stage (Sect. 5.4).

**Fig. 6:**
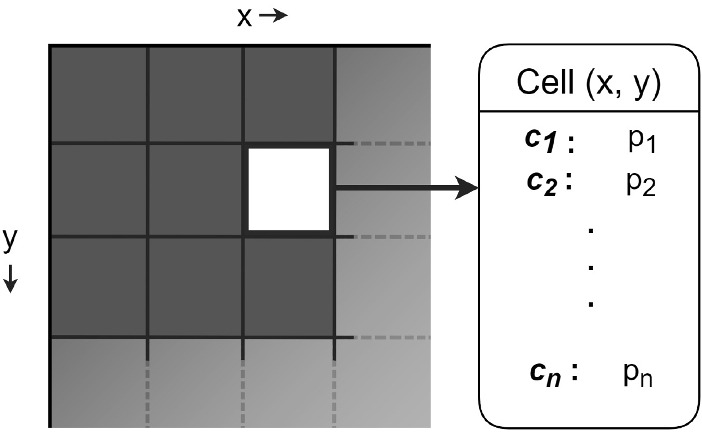
Schematic description of the grid-like structure employed during the opacity film generation procedure. For each cell of the grid, we store the colours (labelled $c_1$ through $c_n$, from a selected set) assigned to the particles placed within that cell as well as the number of particles (labelled $p_1$ through $p_n$) associated with each of the selected colours

To account for the variation in colour that one would expect in a real setting, we consider multiple particles with distinct chromatic attributes falling on the same location at a given timestep. More precisely, we employ a grid-like structure, illustrated in Fig. 6, to track the particles at each location, and select a set of colours for them based on the type of environment being considered. For example, from our observations, we found three shades of grey to be sufficient to depict household dust in most cases. In Sect. 6, we examine such choices more closely.

For simplicity, we can select a grid with dimensions equal to those of the desired texture map, i.e. we can associate each cell with a pixel (on the raster file representing the texture map). Note that it is also feasible to associate a cell with multiple pixels, which could be used to represent larger particles.

We use Eq. 1 to calculate the number of fine particles to be added to a target object’s surface at any given timestep. Subsequently, for each of these particles, we select a random location within the bounds of the corresponding texture map and one of the colours from the predefined colour set. After we determine the grid cell associated with the selected location, we increment the number of particles of the matching colour within that cell. Once all of the particles have been added to the grid, the final particle colour distribution at each cell is employed to calculate the resulting colour of the pixel associated with that cell.

More precisely, to obtain the resulting pixel colour at cell {x, y} and timestep *t*, we use the following sets of equations. We begin by computing the colour $\varvec{u}$ for a given cell as:

2 $$\begin{aligned} \varvec{u}_{x, y, t}&={\sum _{i=1}^n{\frac{p_{x, y, t, i}\varvec{c}_i}{p_{x, y, t}}}}, \end{aligned}$$

where $p_{x, y, t, i}$ represents the number of particles associated with the *i*th colour that have accumulated at the given location and time, $p_{x, y, t}$, represents the total particles accumulated at the given location and time, and $\varvec{c}_i$ represents the *i*th colour.

Afterwards, to adjust the particle opacity, we employ a parameter that represents the adjusted number of particles at a location {x, y} in a given timestep *t*. This parameter, denoted ANP, is computed as:

3 $$\begin{aligned} ANP_{x, y, t}&= \sum {\omega _ip_{x, y, t, i}}, \end{aligned}$$

where $w_i$ represents the selected weight of the *i*th colour. A high weight will result in particles of that colour being more opaque.

Using the parameter ANP, we then compute the relative opacity value, denoted *RO*, for the cell at a timestep *t* as:

4 $$\begin{aligned} RO_{x, y, t}&= \frac{ANP_{x, y, t}}{ANP_{max}}\; \epsilon \; [0, 1], \end{aligned}$$

where the value of $ANP_{max}$ is selected according to the desired appearance. In general, smaller $ANP_{max}$ values will result in an opacity increase for every dust particle in the resulting texture map. This step enables fine control over the overall opacity of the texture map.

Lastly, we calculate the resulting pixel value for our texture map, denoted $\varvec{C_{op}}$, by multiplying our computed colour and opacity values:

5 $$\begin{aligned} {\varvec{C_{op}}}_{x, y, t} = \varvec{u}_{x, y, t}RO_{x, y, t}. \end{aligned}$$

### Granularity mask

Similar to the opacity film, we chose to represent the granularity mask as a texture map and used Eq. 1 to calculate the total number of coarse elements to be added to a target object’s surface at any given timestep. Unlike the opacity film, the particles in the granularity mask vary not only in terms of their chromatic attributes, but also in terms of their size, shape and orientation. Also, based on our visual inspection of real dusty settings, we assume the coarse dust elements to be opaque and place their constituent particles directly on the texture map.

**Fig. 7:**
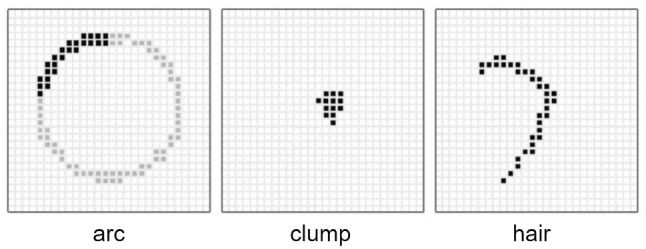
Three example shapes (arc, clump, and hair) that may be used by our granularity mask generation procedure to represent coarse dust elements spanning a number of pixels on the corresponding texture map

For each of the coarse elements, we select a random location within the boundaries of the corresponding texture map to represent the centre point of the shape. Subsequently, the shape representing the coarse dust element is drawn around this centre point. In Fig. 7, we provide a few example shapes that can be employed to represent coarse dust elements, whose generation is outlined in Sects. 5.3.1 to 5.3.3. The output of the selected shape drawing algorithm at a given timestep *t* is then stored as:

6 $$\begin{aligned} b_{x, y, t} = {\left\{ \begin{array}{ll} 1 &{} \text {if \{x, y\} is occupied by a coarse element,} \\ 0 &{} \text {otherwise}. \\ \end{array}\right. } \end{aligned}$$

We can then calculate the resulting pixel (colour) value at each location $\{x,y\}$ on the texture map representing the granularity mask using:

7 $$\begin{aligned} {\varvec{C_g}}_{x, y, t}&= \varvec{k} b_{x, y, t}, \end{aligned}$$

where $\varvec{k}$ is a user-defined colour assigned to the coarse dust elements.

We note that, similar to the fine particles forming an opacity film, it is possible to have distinct colours assigned to coarse elements forming a granularity mask. In that case, the colour associated with a given pixel could also be specifically or randomly selected from a set of colours previously defined by the user. Again, from our visual inspections of real dusty settings, we found that a single colour is usually sufficient to obtain plausible dust appearance depictions. Thus, although Eq. 7 can be modified to account for the assignment of different colours to coarse dust elements, it is provided in this present form for practicality. We illustrate this aspect in Sect. 6.

In the remainder of the section, we outline the procedures employed to describe the distinct shapes, namely arcs, clumps and hairs, used to represent the coarse elements. For each of the selected shape descriptions, *rand* corresponds to random number generator with a range between 0 and 1 (inclusive).

#### Arcs

Our implementation of arcs is indicated in the pseudocode provided in Algorithm 1. In the proposed procedure, we employ a configuration parameter *r* to represent the radius (in pixels) of the underlying circle, an angle $\phi$ to represent the desired arc length, and recommend an integer value *n* equal to approximately $2\pi r$ to avoid gaps in the rasterized output. 
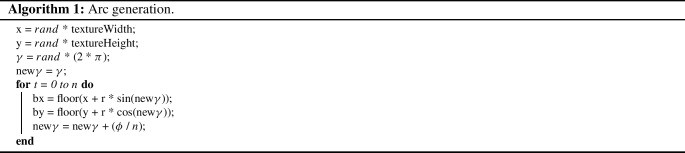


#### Clumps

From our observations, we found that a simple clump implementation consisting of a number of particles *p*, randomly spread over a rectangular area with height $w_y$ and width $w_x$, resulted in clumps with a believable appearance. We provide the pseudocode for the generation of such clumps in Algorithm 2. 
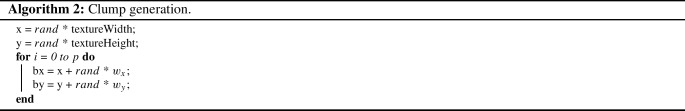


#### Hairs

For the more complex hair elements, we employed three-point Bèzier curves, drawn using the De Casteljau’s procedure [17]. In our application of this procedure, whose pseudo-code is outlined in Algorithm 2, we employ a configuration parameter *w* to represent the maximum width of the generated coarse element, with a larger *w* value resulting in more noticeable elements (on average). 
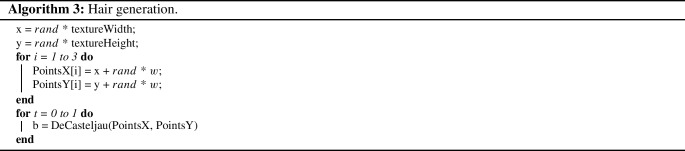


### Applying the texture maps to dusty scenes

After generating the texture maps for the opacity film and granularity mask, one can proceed to render scenes depicting dusty objects. To realistically represent their appearance using texture maps, however, one should also account for the effects resulting from variations in the viewing angle $\theta _v$ and the light incidence angle $\theta _i$ (Sect. 3).

To account for the effect of $\theta _v$ variations on the dusty appearance at a point $\{x, y\}$ on a target object, we employ a parameter expressed as:

8 $$\begin{aligned} V_{x, y}&= 1 - (cos\; \theta _{v_{x, y}}), \text {for} 0^{\circ } \le \theta _{v_{x, y}} \le 90^{\circ }. \end{aligned}$$

Similarly, to account for the effect of $\theta _i$ variations, we employ the following parameter:

9 $$\begin{aligned} L_{x, y}&= 1 - \frac{1}{2}(cos\; \theta _{i_{x, y}}), \text {for}0^{\circ } \le \theta _{i_{x, y}} \le 90^{\circ }. \end{aligned}$$

We note that, although the presence of dust elements becomes less noticeable when one considers $\theta _i=0^{\circ }$ (Sect. 3), they can still be perceived. This aspect can be verified in the photographs presented in Fig. 2. Hence, we elected to reduce the weight of the $cos\; \theta _i$ term by half based on our empirical observations (Sect. 3).

We can then represent the appearance of the dusty objects in a given scene using the following equations to calculate the colour associated with each point $\{x, y\}$ on their surfaces at a timestep *t*:

- If $${\varvec{C_g}}_{x,y,y} \ne (0, 0, 0)$$, then
  10 $$\begin{aligned} \varvec{C}_{x, y, t} = {\varvec{C_g}}_{x,y,t} L _{x,y}+ \varvec{M}_{x,y}(1 - L_{x,y}), \end{aligned}$$
  where $\varvec{M}_{x,y}$ represents the colour of a target object’s substrate surface at the point $\{x, y\}$.
- Otherwise
  11 $$\begin{aligned} \varvec{C}_{x, y, t} ={\varvec{C_{op}}}_{x,y,t} V_{x,y}L_{x,y} + \varvec{M}_{x,y}(1 - V_{x,y} L_{x,y}).\nonumber \\ \end{aligned}$$

**Fig. 8:**
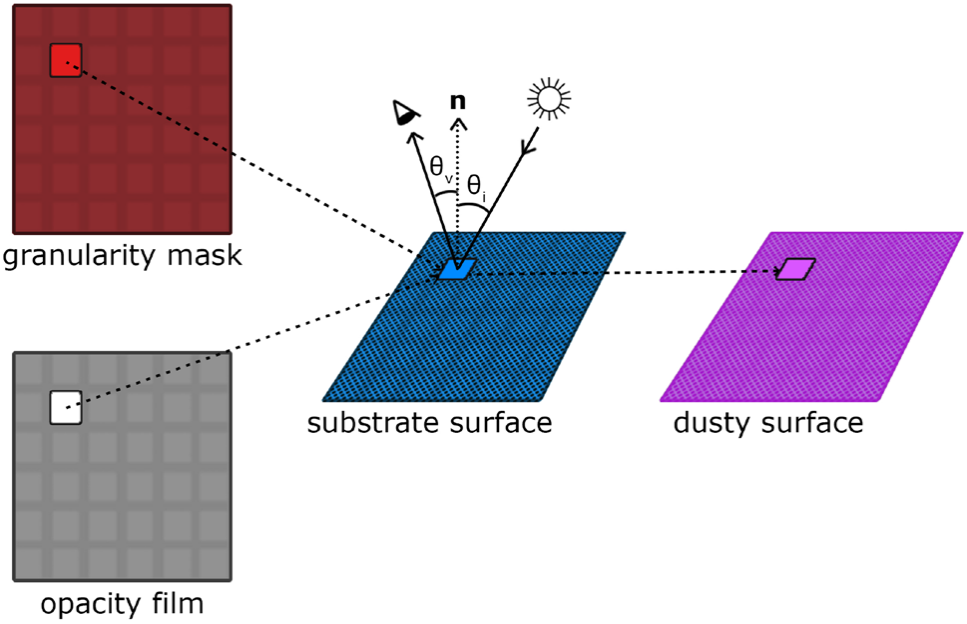
A visual representation of the final stage of proposed framework employed in the rendering of a dusty surface. After obtaining the texture maps representing the opacity film and granularity mask, we determine the resulting pixel values for a dusty surface taking into account the viewing and light incidence angles, represented by $\theta _v$ and $\theta _i$, respectively, and measured with respect to the surface’s normal vector $\varvec{n}$

In Fig. 8, we provide a visual representation of this final calculation, showing the texture maps and geometric information used by the proposed framework.

To further enhance the perceived degree of realism of certain rendered scenes (e.g. depicting curves objects), we found it beneficial to incorporate to the proposed framework a spatial falloff computation step based on the work of Hsu and Wong [25]. For that purpose, we consider a dust source placed directly above a target object and employ the *z*-component of the object’s normal vector at the point of interest to determine the strength of dust falloff at this point. More precisely, we begin by mapping the *z* component to values between 0 and 1, which will be used to determine the strength of the spatial falloff. We then multiply the resulting mapped value, denoted $z_m$, by the dust colour value $\varvec{C}_{x, y, t}$, and (1 - $z_m$) by the material colour value $\varvec{M}_{x,y}$, to obtain the target object’s adjusted dusty appearance at the point of interest.

For scenes depicting objects with crevices and/or objects in relatively close proximity (e.g. the block of sticky notes and the lamp shown in Fig. 1c), we also found it beneficial to take into account ambient occlusion [34]. We note, however, that this procedure can add an additional level of plausibility at the expense of a higher computational cost. Similar to the application of the dust falloff technique mentioned before, one can multiply the resulting ambient occlusion factor (between 0 and 1) by the colour value obtained using Eqs. 10 or 11.

## Results and discussion

In this section, to showcase the applicability of the proposed framework, we present five test scenes, henceforth referred to as paper clip, wood pieces, teapot, sphere and desktop. We also highlight key aspects associated with the appearance of the depicted dusty objects and indicate possible extensions to the proposed framework.

For all test scenes, we found the following three colour shades (in RGB format) to be sufficient for representing the fine particles forming the opacity films: dark grey (100, 100, 100), medium grey (150, 150, 150) and light grey (200, 200, 200). For the granularity masks, we elected to use a single colour (220, 220, 220) to be assigned to their coarse dust elements.

We considered the placement of each dust element to be equally likely across the entire object and used a uniform random distribution accordingly. During the generation of the texture maps, we employed a single pixel (on the respective map) to represent a fine particle and found a resolution of 150 pixels per *in* appropriate for this purpose.

Furthermore, we used $ANP_{max}$ equal to 1.2 in all test scenes and varied the values assigned to the *A* and *w* parameters (Table 1) used in the generation of their respective texture maps. Lastly, our selection of values for the $\alpha$ and $\beta$ dust accumulation parameters employed in each scene took into consideration the outcomes of the observational experiments described in Sect. 4. These values, along with the level(s) of human presence and activity associated with each scene, are also provided along with the results’ presentation.

**Table 1:** Values assigned to the framework parameters *A* (target surface area) and *w* (coarse element maximum width) used in the generation of the textures employed in the rendering of the test scenes

Scene	*A* ($in^2$)	*w* (pixels)
paper clip	146	30
wood pieces	100	12
teapot	1600	24
sphere	4000	14
desktop	814	36

To obtain the images presented this section, we implemented the proposed framework in C++ and ran it on a computer with a GTX 1070 GPU and Ryzen 9 3950X CPU. To render the scenes depicted in these images, we used Blender’s cycles path tracing feature with a custom Node setup according to Eqs. 9 to 11. It is worth mentioning that the generation of the dust textures at 150 ppi for the largest test scene averaged 70 seconds. The rendering of this scene, which is depicted in Fig. 10 (f), involved the placement of 1,187,200 fine particles and 551,200 coarse elements.

**Fig. 9:**
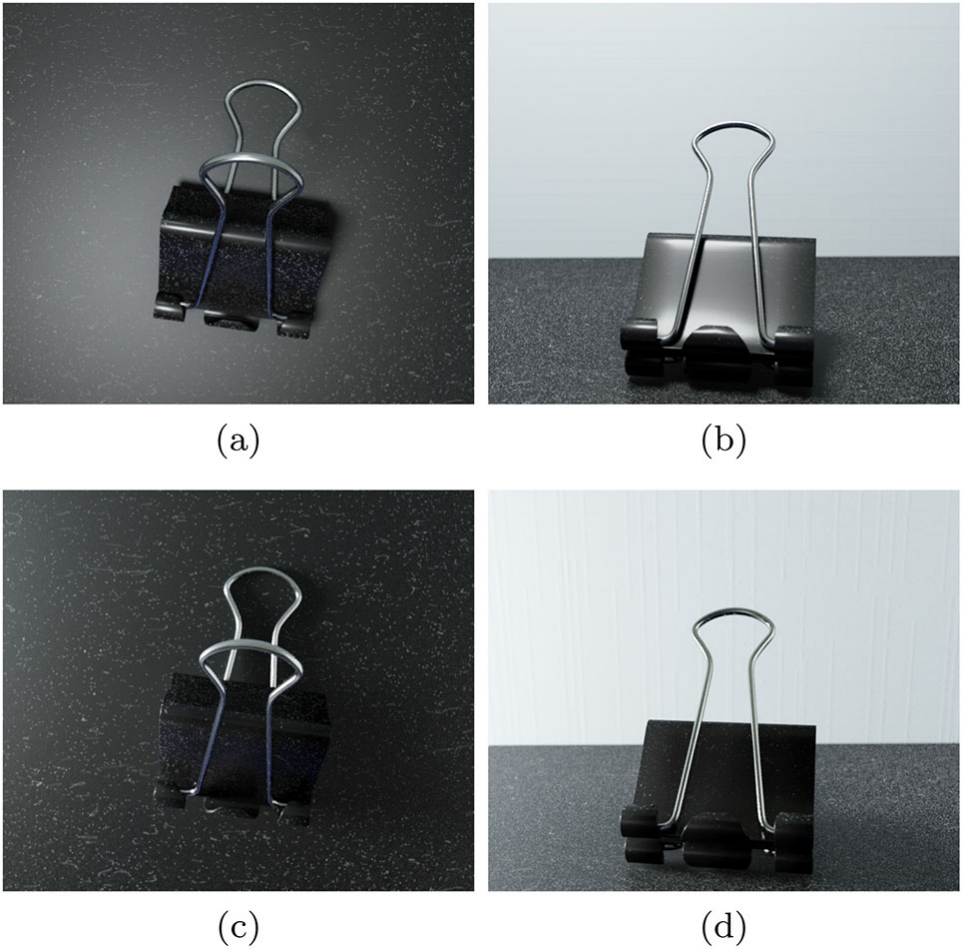
Images of a dusty scene generated considering distinct viewing ($\theta _v$) and light incidence ($\theta _i$) angles measured with respect to the flat surface’s normal vector. **a** $\theta _v=0^{\circ }$ and $\theta _i=0^{\circ }$. **b** $\theta _v=80^{\circ }$ and $\theta _i=0^{\circ }$. **c** $\theta _v=0^{\circ }$ and $\theta _i=80^{\circ }$. **d** $\theta _v=80^{\circ }$ and $\theta _i=80^{\circ }$

**Fig. 10:**
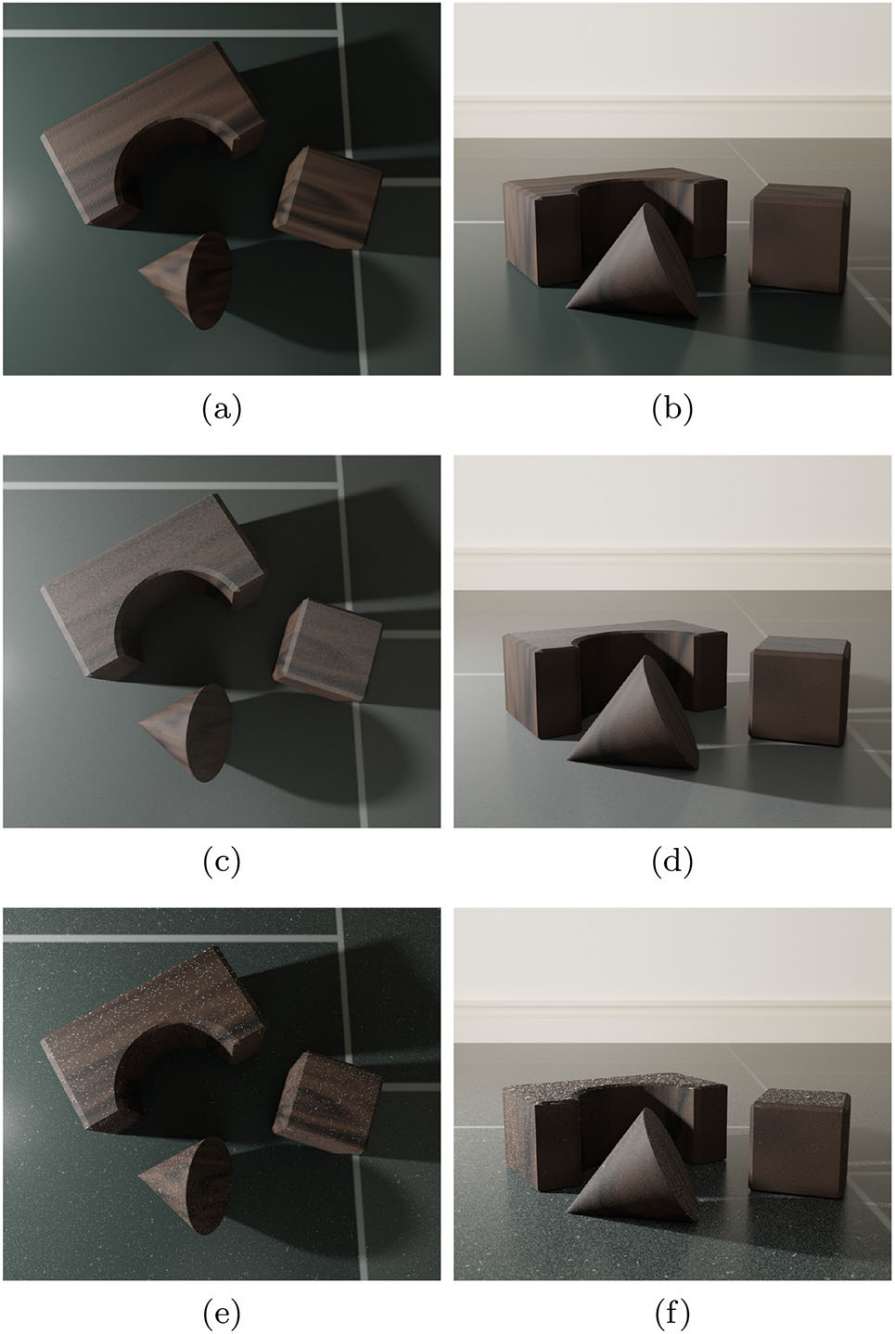
Images of a scene without (top row) and with accumulated dust. The dust representations in the latter were generated using a well-known physically inspired method [25] (middle row) and the proposed framework (bottom row). Two distinct viewing ($\theta _v$) angles were considered: $\theta _v=10^{\circ }$ (left column) and $\theta _v=80^{\circ }$ (right column)

### Rendered scenes

Initially, we employed the paper clip scene (Fig. 9) to examine the effects of varying viewing and illumination geometries on the appearance of dusty objects (Fig. 2), notably after an extended period of dust accumulation. This involved the generation of images using two distinct values for the viewing and light incidence angles, namely $0^{\circ }$ and $80^{\circ }$. To obtain the texture maps, we considered values for the $\alpha$ and $\beta$ parameters appropriate for representing an environment characterized by a low level of human presence and activity. More specifically, for the opacity film, we used $\alpha$ and $\beta$ values equal to 2.4 and 0.8 dust elements per $in^2$, respectively, while for the granularity mask, we used $\alpha$ and $\beta$ values equal to 0.1 and 0 dust elements per $in^2$, respectively. Upon visual inspection of the resulting images, one can observe that, as expected (Sect. 3), the opacity film becomes more noticeable as the viewing angle is increased (Fig. 9b, d). Similarly, the combined effects of the opacity film and granularity mask appear most noticeable when the light incidence angle is increased (Fig. 9d).

**Fig. 11:**
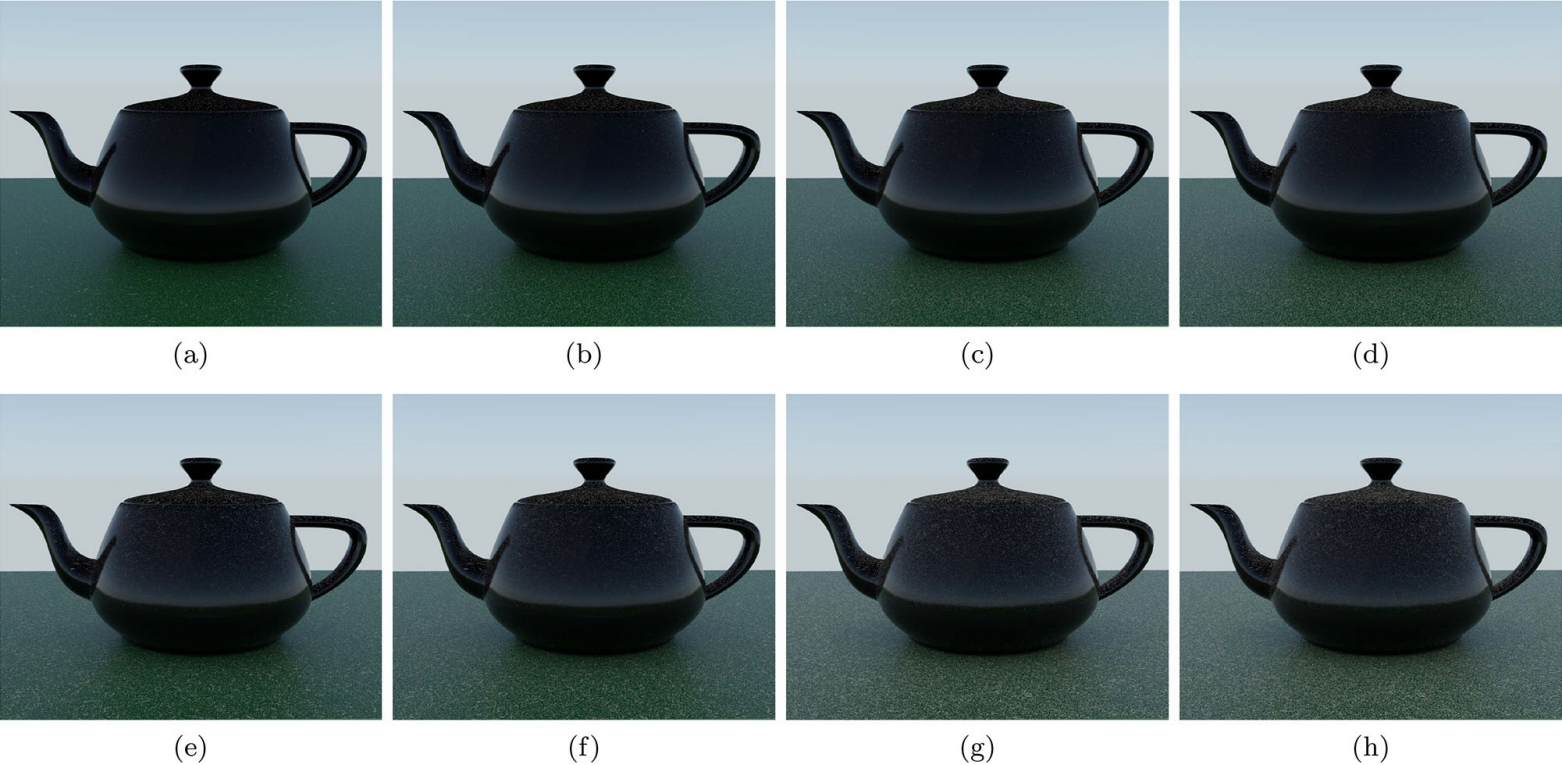
Images of a dusty scene depicting dust accumulation over time in environments with low (top row) and high (bottom row) levels of human presence and activity. **a** and **e**: after 100 days of dust accumulation. **b** and **f**: after 200 days of dust accumulation. **c** and **g**: after 300 days of dust accumulation. **d** and **h**: after 400 days of dust accumulation

We note that dust appearance variations elicited by changes in the illumination and viewing geometries (Fig. 2) become more noticeable when one accounts for the distinct roughness levels of dust layers. To further illustrate this aspect, in Fig. 10, we presented images obtained for the wood pieces scene using a well-known physically inspired method [25] as well as images generated using our proposed method, with the opacity film’s $\alpha$ and $\beta$ values set to 2.4 and 0.8 dust elements per $in^2$, respectively, and the granularity mask’s $\alpha$ and $\beta$ values set to 0.1 and 0 dust elements per $in^2$, respectively. As it can be observed in the images presented in Fig. 10c and d, changes in the viewing angle have a negligible impact on the appearance of the deposited dust layer when distinct levels of roughness are not taken into account. Conversely, as it can be observed in the images depicted in Fig. 10e and f, changes in the viewing angle result in the expected appearance variations when these levels are accounted for.

We then used the teapot scene (Fig. 11) to examine the changes in the accumulation of dust over time in environments with different levels of human presence and activity. The images presented in Fig. 11a–d were rendered considering a low level of human presence and activity. Accordingly, for the opacity film, we used $\alpha$ and $\beta$ values equal to 2.4 and 0.8 dust elements per $in^2$, respectively, while for the granularity mask, we used $\alpha$ and $\beta$ values equal to 0.1 and 0 dust elements per $in^2$, respectively. The images presented in Fig. 11e–h, on the other hand, were rendered considering a high level of human presence and activity. Thus, for the opacity film, we used $\alpha$ and $\beta$ values equal to 2.8 and 25.7 dust elements per $in^2$, respectively, while for the granularity mask, we used $\alpha$ and $\beta$ values equal to 1.3 and 0.3 dust elements per $in^2$, respectively. In the rendering of the images presented in Fig. 11, we also employed the spatial falloff technique outlined in Sect. 5.4 to improve the appearance of dust accumulated on the teapot’s surface. As expected, the accumulation of dust is markedly more noticeable in the images generated considering an environment characterized by a high level of human presence and activity (Fig. 11e–h), particularly with respect to the presence of coarse dust elements.

**Fig. 12:**
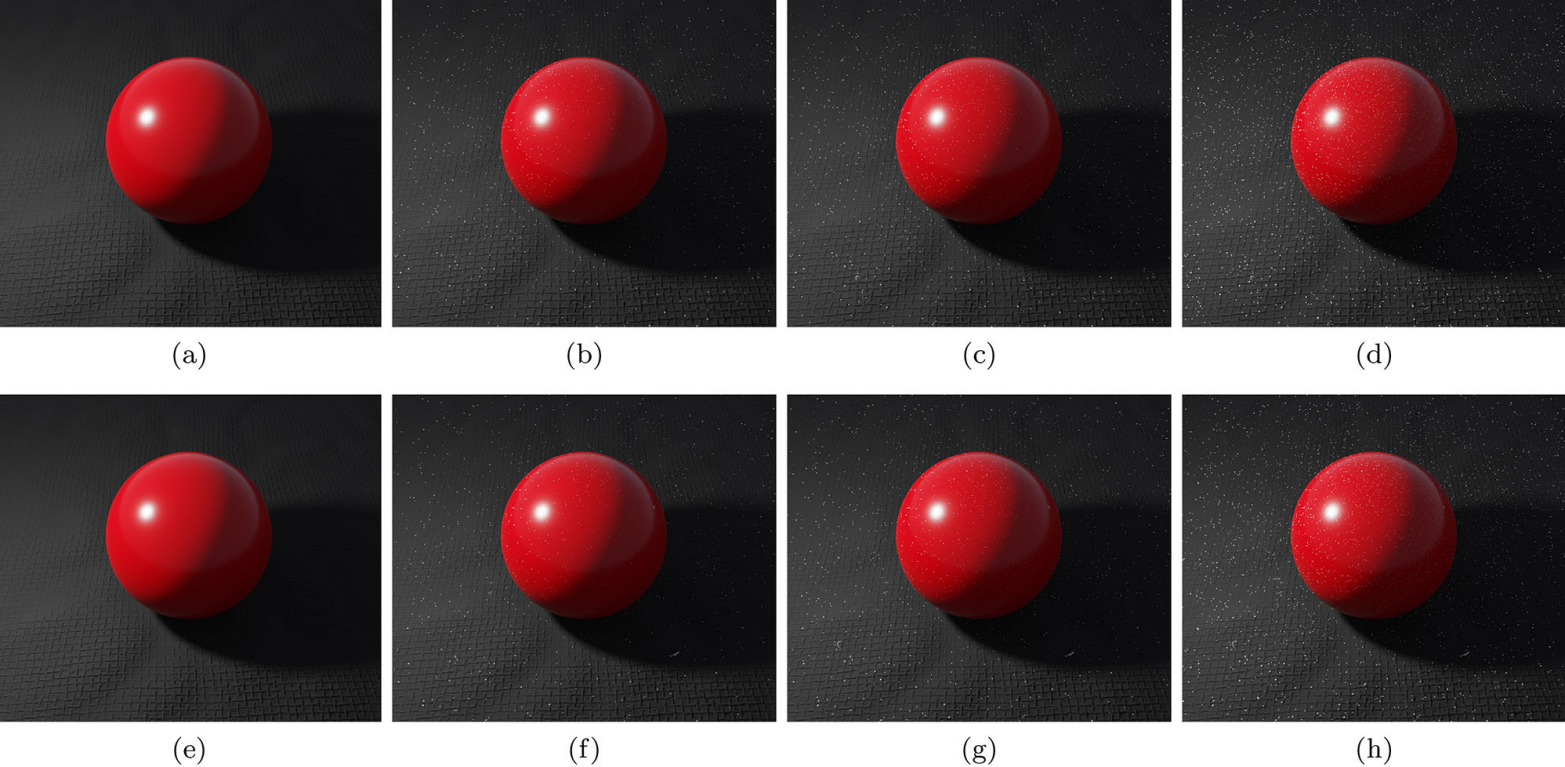
Images of a dusty scene depicting dust accumulation over time considering distinct accumulation rates. **a** and **e**: initial state. **b** and **f**: after 5 days of dust accumulation. **c** and **g**: after 10 days of dust accumulation. **d** and **h**: after 100 days of dust accumulation. For the sequence in the top row, we employed a steeper accumulation rate during the first 5 days, and a lower rate during the remaining days. For the sequence in the bottom row, for comparison purposes, we employed the lower rate for all 20 days

**Fig. 13:**
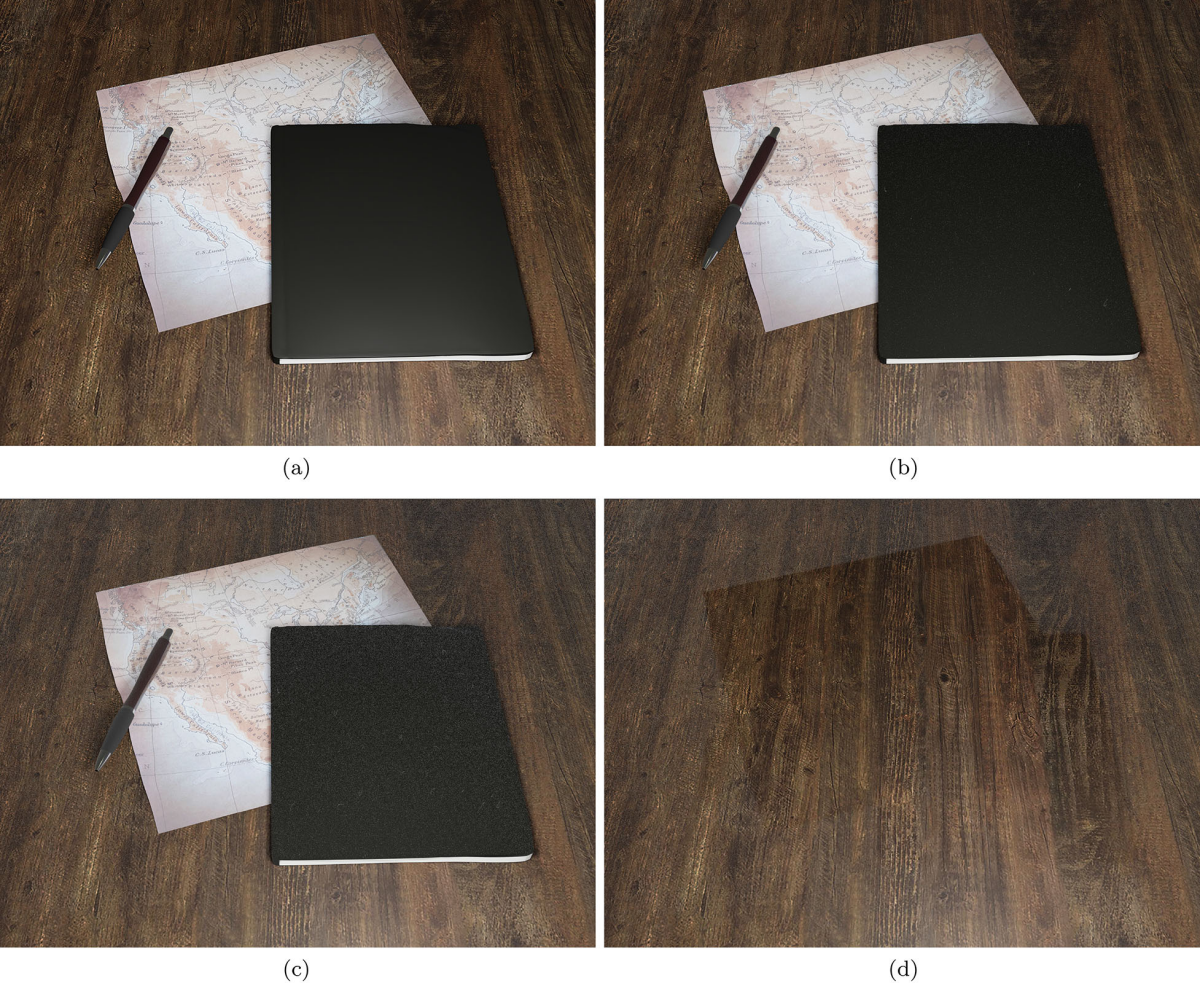
Images illustrating the accumulation of dust in a virtual setting over time. **a** Clean surfaces. **b** Surfaces after 60 days. **c** Surfaces after 365 days. **d** Relatively dust free areas resulting from the removal of objects after 365 days of dust accumulation

Recall that the dust accumulation rate can vary in a given environment, notably with respect to the deposition of fine particles, under certain circumstances (Sect. 4, Fig. 5c). As we suggested earlier, situations like that could be potentially addressed by applying distinct versions of Eq. 1, i.e. with distinct values assigned to the parameters $\alpha$ and $\beta$, in a piecewise fashion. To illustrate this possibility, we employed this strategy to the opacity film textures used in the rendering of the sphere scene images (Fig. 12). In the initial five days (Fig. 12 a and b), we considered a steeper rate with $\alpha$ and $\beta$ values equal to 11.1 and 5.1 dust elements per $in^2$, respectively, while for the remaining days (Fig. 12 c and d), we considered a slower rate with $\alpha$ and $\beta$ values equal to 2.8 and 25.7 dust elements per $in^2$, respectively. As for the granularity masks, we used $\alpha$ and $\beta$ values equal to 1.3 and 0.3 dust elements per $in^2$, respectively. For comparison purposes, we also rendered images obtained considering only the slower accumulation rate for the fine dust particles during the entire dust accumulation period (Fig. 12 (bottom row)). As expected, a piecewise application of Eq. 1 can approximate situations like that depicted in Fig. 5c more closely from a numerical standpoint. Nonetheless, it is also worth noting that, from a visual inspection standpoint, the differences in comparison with a single application of Eq. 1 may not be quite significant depending on the values selected for the parameters $\alpha$ and $\beta$.

Lastly, we employed the desktop scene (Fig. 13) to further illustrate the use of the proposed framework in the rendering of believable images depicting changes in the appearance of dusty objects over time. There may be situations in which the level of human presence and activity, combined with distinct air circulation conditions, may affect the accumulation of dust elements in different ways. Thus, for this scene, we elected to consider markedly distinct accumulation rates for the fine particles and coarse dust elements. Accordingly, for the opacity film, we used $\alpha$ and $\beta$ values equal to 10 and 25.7 dust elements per $in^2$, respectively, while for the granularity mask, we used $\alpha$ and $\beta$ values equal to 0.1 and 0 dust elements per $in^2$, respectively. The provided sequence of images shows the objects in an initial (“clean”) state (Fig. 13a), and after dust accumulation periods of 60 days (Fig. 13b) and 365 days (Fig. 13c). We also applied the spatial falloff technique in conjunction with ambient occlusion, both mentioned in Sect. 5.4, to represent dust deposits in areas in which the presence of some objects (e.g. the pen and the notebook) can directly affect the accumulation of dust on others (e.g. the desktop). The resulting subtle effects can be observed in the image depicting the removal of the objects on the desktop after 365 days of dust accumulation (Fig. 13d).

It has been recognized that material appearance perception is a challenging problem for synthetic, pristine objects, even when these objects are subjected to controlled viewing and illumination conditions [11]. Although environmental factors, such as the presence of dust, can provide important visual cues and lead to an increase in the perceived level of realism of these objects, from an optics perspective, they may add yet another layer of complexity to the problem. Certainly, for applications demanding predictive images, it would be appropriate to employ a physically based framework for the simulation of dust accumulation over time. We remark, however, that the proposed framework was designed using a physically inspired approach and aiming at the cost-effective generation of plausible images of dusty objects. The sequences of images presented in this section indicate that the proposed observation-driven framework can fulfill this objective. Nonetheless, we believe that there is still room for enhancements. We briefly address this aspect next.

### Extensions

The proposed framework can be extended in different ways. For conciseness, in the remainder of this section, we will outline a few of particular interest, which we intend to explore in our future research.

The first extension would involve providing support for handling sporadic disturbances on the accumulated dust within a given scene. To achieve that, one could define a disturbance function that would remove particles from the grid representing the opacity film, or remove coarse elements from the granularity layer from one timestep to the next. This would mimic processes such as wind gusts or direct manipulations that remove dust elements from a location as they accumulate. It is worth mentioning that the modelling of changes in the appearance of granular materials, such as sand and snow [14, 16, 42], caused by external forces is an active area of research. Although similar concepts could be potentially applied to dust layers, such an undertaking would merit an investigation on its own right.

A second extension of interest would involve the representation of the particles of the granularity mask as geometric objects rather than as a texture map. We note, however, that although this approach could make the coarse elements more noticeable, it would likely incur significantly higher computational costs.

Lastly, another extension would focus on streamlining the process of sizing the resulting dust texture maps. This could involve a method for generating the textures at multiple resolutions for use in conjunction with the texture level of detail algorithms [2, 19]. This would be beneficial for scenes involving a large number of objects, where there may be a prohibitive amount of work involved in manually determining the correct texture file sizes to maintain a consistent scale for the simulated dust particles.

## Conclusion and future work

We have proposed a physically inspired framework for the generation of textures depicting temporal variations on dust layers covering objects composing virtual settings. Its design is based on the representation of this ubiquitous material using two abstract levels of roughness, referred to as the opacity film and the granularity mask. The texture maps associated with these two representations are then combined during rendering taking into account the viewing and illumination geometries employed to obtain the target materials’ depictions.

In this paper, we have also described observational experiments that provided the insights leading to the proposed representation of dust layers. Moreover, these experiments allowed us to gather qualitative and quantitative information about time-dependent changes on these layers, particularly due to different levels of human presence and activity in the environments under inspection. This information was then incorporated into our texture generation algorithms. The rendered sequences presented to illustrate the applicability of the proposed framework indicate that it can be effectively employed to obtain plausible depictions of changes in the appearance of dusty objects over time.

The perceived appearance of real materials is often affected by environmental factors. The accumulation of dust over time is among the most prevalent of these factors. Accordingly, a number of works based on different physically based and physically inspired approaches have been proposed to account for these factors and, consequently, increase the degree of realism of synthetic scenes. Viewed in this context, the outcomes of this research can be seen as tangible contributions to these efforts, notably with respect to the generation of believable images of dusty objects at a relatively low computational cost.

As future work, besides addressing the aforementioned possible extensions to the proposed framework, we plan to extend our observational experiments to a wider variety of environments and dust compositions (e.g. dust originating from wind-transported sand). We also intend to investigate alternatives to streamline the application of dust textures in complex scenes and explore their use in conjunction with first-principles material appearance models in order to enhance the fidelity-to-cost ratio of predictive renderings of dusty objects. The development of such models couple with the simulation of external factors, such as static electricity and friction, affecting dust deposition patterns would also represent interesting avenues for future research in this area.
